# A Novel Thermal-Activated β-Galactosidase from *Bacillus aryabhattai* GEL-09 for Lactose Hydrolysis in Milk

**DOI:** 10.3390/foods11030372

**Published:** 2022-01-27

**Authors:** Shuyue Luan, Xuguo Duan

**Affiliations:** Department of Food Science and Technology, College of Light Industry and Food Engineering, Nanjing Forestry University, Nanjing 210037, China; luanshuyue@njfu.edu.cn

**Keywords:** β-galactosidase, *Bacillus aryabhattai*, thermal-activated, lactose hydrolysis, milk

## Abstract

β-Galactosidase has been greatly used in the dairy industry. This study investigated a novel thermostable β-galactosidase (lacZBa) from *Bacillus aryabhattai* GEL-09 and evaluated the hydrolytic performance of this enzyme. Firstly, the lacZBa-encoding gene was cloned and overexpressed in *Escherichia coli* BL21(DE3). Phylogenetic analyses revealed that lacZBa belonged to the glycoside hydrolase family 42. Using SDS-PAGE, we determined that the molecular weight of lacZBa was ~75 kDa. Purified lacZBa exhibited a maximum activity at 45 °C, pH 6.0, and could be activated following incubation at 45 °C for several minutes. The half-life of lacZBa at 45 °C and 50 °C was 264 h and 36 h, respectively. While Co^2+^, Mn^2+^, Zn^2+^, Fe^2+^, Mg^2+^, and Ca^2+^ enhanced enzymatic activity, Cu^2+^ and ethylenediaminetetraacetic acid inhibited enzymatic activity. Moreover, lacZBa could hydrolyze lactose and oNPG with *K*_m_ values of 85.09 and 14.38 mM. Molecular docking results revealed that lacZBa efficiently recognized and catalyzed lactose. Additionally, the hydrolysis of lactose by lacZBa was studied in lactose solution and commercial milk. Lactose was completely hydrolyzed within 4 h with 8 U/mL of lacZBa at 45 °C. These results suggested that lacZBa identified in this study has potential applications in the dairy industry.

## 1. Introduction

β-Galactosidase (EC 3.2.1.23) naturally catalyzes the hydrolysis of β-1,4 galactosyl bonds of lactose, yielding glucose and galactose, which are kinds of glycoside hydrolase that play a significant function in the dairy industry. It can be used to yield dairy products and lactose-free milk. Lactose-free milk is suitable for people with lactose intolerance, which is prevalent in more than 70% of the world’s adult population, and more than 90% among East Asians [1]. Meanwhile, lactose-free milk is also beneficial for acid formation and lactic acid content in yogurt. Additionally, due to the low solubility of lactose, it is easy to crystallize in condensed milk products such as condensed milk and ice cream, affecting the flavor and quality of the products. Moreover, when lactose is hydrolyzed to a monosaccharide, the hydrolysis of lactose minimizes crystallization in sugar-containing foods, thereby avoiding grittiness [2]. Therefore, it is of practical significance to develop new types of β-galactosidase and study the hydrolysis of lactose in milk by β-galactosidase in order to improve the national diet structure, giving full play to the health functions of milk.

β-Galactosidases are ubiquitous enzymes produced by animals, plants, and microorganisms. In animals, β-galactosidases exist in the intestine [3] and milk of mammals. In plants, they mainly exist in stems, leaves (including cytoplasm and cell wall) and seeds. Additionally, β-galactosidases exist in a wide range of microorganisms, including bacteria, molds, yeasts, etc. The source bacteria include *Lactobacillus* [4,5], *Bifidobacterium* [6], *Escherichia coli* [7], *Bacillus* sp. [8,9,10]. The source molds include *Aspergillus oryzae* [11], *Aspergillus niger* [12,13], and the source yeasts include *Kluyveromyces lactis* [14], *Kluyveromyces fragilis* [15], etc. Based on protein sequence classification and evolutionary relationships, β-galactosidases can be classified into four distinct glycoside hydrolase (GH) families, GH-l, GH-2, GH-35, and GH-42, showing different catalytic and structural characteristics [16]. All the members of GH-42 are β-galactosidases. Most of them come from extremophiles, so they usually have some special properties. Several GH-42 β-galactosidases have been identified and heterologously expressed. For example, the β-galactosidase from *Geobacillus stearothermophilus* is thermostable, and its optimum temperature is 70 °C [17]. Moreover, *Arthrobacter* sp. ON14, isolated from Antarctica, exhibits high catalytic activity at 15 °C [18]. β-Galactosidase from *Exiguobacterium acetylicum* can maintain 80% residual enzyme activity at 40 °C for 200 h and has good thermostability [19]. Furthermore, the structures of GH-42 β-galactosidases have also been described in theoretical studies. The members of GH-42 have typical TIM barrels, which consist of eight repeating β-fold-joined α-helix forming the catalytic domain of the enzymes [20].

β-Galactosidases have been isolated from different sources, such as microorganisms, animals, and plants. However, the most widely used enzymes are microbial β-galactosidases [21]. The relative molecular weight, pH value, temperature and other biochemical properties of β-galactosidases vary greatly with the microbial sources. So far, β-galactosidase has been used for the hydrolysis of lactoses, like milk and cheese whey. A fraction of microbial β-galactosidases have an optimal pH between 2.5 and 5.4, and are chiefly applied for acidic whey hydrolysis [22]. In contrast, other β-galactosidases of microbial origin have the highest activity between pH 6.0 and 7.0, and these are more suited to the hydrolysis of sweet whey and milk [23,24]. In addition, thermostability also affects the ability of β-galactosidase to hydrolyze lactose. In addition, different microbial sources of β-galactosidases have different thermostability. Good thermostability is one of the most significant characteristics of enzymes. In industrial production, thermostable β-galactosidases have the advantages of wide application range and high conversion rate. Additionally, higher temperatures can raise the enzyme’s initial productivity, provide substrates with higher solubility, and prevent microbial contamination [25]. At present, the β-galactosidases used to hydrolyze lactose in the dairy industry mostly come from yeast, *Aspergillus oryzae*, *Aspergillus niger* or *Lactobacillus*. However, most of them have the disadvantage of poor thermostability [26].

*Bacillus aryabhattai* Gel-09 (CCTCC M2017320), a newly isolated raw starch-digesting amylase-producing strain, was screened and identified in our laboratory [27]. After further analysis of the genome of *B. aryabhattai* Gel-09, we found that a protein encoded in the genome of this strain may be a new β-galactosidase (lacZBa). We expect that the new protein will have different properties from the existing β-galactosidases, which will provide value for future scientific research or commercial production. In this study, the β-galactosidase-encoding gene of *B. aryabhattai* GEL-09 was cloned and heterologously expressed in *Escherichia coli* BL21(DE3), and the recombinant enzyme was characterized in detail. Furthermore, the hydrolysis of lactose by lacZBa was studied in lactose solution and commercial milk.

## 2. Materials and Methods

### 2.1. Bacterial Strains and Plasmid Vectors

*Bacillus aryabhattai* GEL-09, newly isolated by our laboratory, has been deposited in the China General Microbiological Culture Collection Center (CCTCC, Wuhan, China) under accession No. M2017320 [27]. *E. coli* JM109 was used as host for gene cloning, and *E. coli* BL21(DE3) was used for the production of recombinant protein. The pET24a was used as the expression vector.

### 2.2. Enzymes and Reagents

Restriction enzymes (*Eco*RI), r*Taq*, DNA standards, DNA fragment recovery kit, genomic DNA isolation kit, and plasmid extraction kit were purchased from Sangong Biotech (Shanghai, China). PCR primers were synthesized by Takara Bio Inc. (Dalian, China). 2-nitrophenyl-β-D-galactopyranoside (oNPG) was obtained from J & K Scientific (Shanghai, China). All other chemicals were purchased from Nanjing Chemical Reagent Co., Ltd. (Nanjing, China) unless otherwise indicated.

### 2.3. Construction of Recombinant Plasmid and Transformation

The *B. aryabhattai* GEL-09 genomic DNA was extracted by using the genomic DNA isolation kit (Sangong Biotech). Two primers were designed (lacZBa-Forword: 5′-GAAGGAGATATACATATGTACATCGGCGTCGATT-3′ and lacZBa-Reverse: 5′-TTGTCGACGGAGCTCCTACATGTTTTCTACAACTCGACT-3′) for amplification. The lacZBa gene was amplified by PCR, and the PCR products were ligated into the pET24a vector digested by *Eco*R I. The ligation mixture was transformed *E. coli* JM109, followed by DNA sequencing of recombinant plasmid. Then, the confirmed plasmid (pET24a-lacZBa) was transformed *E. coli* BL21(DE3) for expression.

### 2.4. Expression and Purification of Recombinant lacZBa in E. coli BL21(DE3)

LB broth, which contained kanamycin (30 μg/mL), was used to culture transformed *E. coli* BL21(DE3) single-colony cells, the cells were cultivated for 8 h at 37 °C. The cells were then cultured for 36 h at 30 °C in TB medium to express lacZBa. The bacteria were collected, suspended in pH 6.0 phosphate buffer, and broken by ultrasonic cell crusher. The supernatant and sediment of recombinant lacZBa were separated under centrifugal conditions for 20 min at 8000 rpm, and the supernatant fraction of recombinant lacZBa was collected and purified using a prepacked DEAE-μSphere ion-exchange column (Tianyan Biotechnology Co., Ltd. Wuxi, China). The purity of recombinant lacZBa was appraised by 12% sodium dodecyl sulfate polyacrylamide gel [28].

### 2.5. β-Galactosidase Activity Assay

Typically, the measurement of β-galactosidase activity using oNPG as the substrates [29]. Firstly, 0.1 mL of diluted enzyme solution was added to 1.8 mL pH 6.0 100 mM sodium phosphate buffer and incubated for 5 min at 45 °C. Then 0.1 mL 20 mM oNPG was added, and the mixture was further incubated for 10 min, after which 1 mL of 1 M Na_2_CO_3_ solution was used to stop the reaction, and absorbance was determined at 420 nm. One unit is the amount of β-galactosidase that catalysis the hydrolysis of 1 μM of oNPG per minute.

### 2.6. Effect of pH and Temperature on lacZBa Activity

The optimal pH of lacZBa was measured over a pH range of 4.5 to 8.0 at 45 °C using sodium phosphate buffer (100 mM), and the pH stability was determined by incubation in the same pH ranges at 4 °C for 24 h. The optimal temperature for β-galactosidase was determined in 100 mM sodium phosphate buffer (pH 6.0) from 30 °C to 55 °C. To study the thermostability of lacZBa, the purified lacZBa was incubated at 45 °C and 50 °C, and the residual lacZBa activity was measured. The initial lacZBa activity was considered as 100% [28].

### 2.7. Effect of Metal Ions on lacZBa Activity

Seven metallic divalent cations (Mn^2+^, Zn^2+^, Cu^2+^, Co^2+^, Fe^2+^, Mg^2+^, and Ca^2+^) and disodium EDTA at final concentrations of 1 mM and 5 mM were added, respectively, and then reacted at 45 °C for 10 min [30]. The lacZBa activity measured in the system without metal ions and chelating agents was regarded as 100%.

### 2.8. Kinetic Parameters of lacZBa

Different concentrations (1–40 mM) and (10–2000 mM) of oNPG and lactose were used as substrates to measure the kinetic parameters of lacZBa, respectively. The reactions were performed in 100 mM sodium acetate buffer (pH 6.0) at 45 °C for 10 min. The kinetic parameters (*K*_m_ and *V*_max_ values) were calculated using Graphpad Prism8 (San Diego, CA, USA). [31].

### 2.9. Hydrolysis of Lactose in Lactose Solution and Commercial Milk

Briefly, 1 mL of purified lacZBa (8 U/mL) was added into lactose solution (containing 50 g/L lactose substrate), and incubated at 45 °C for various times (0, 40, 60, 120, 240 and 480 min). Then, thin-layer chromatography analysis on a silica gel plate (Ocean Chemical Factory, Qingdao, China) was used to analyze the reaction mixture. The mixture n-butanol/HAc/water/ methanol (5:14:1:2, *v/v*) reagents was used as mobile phase. The gel plate was stained by spraying with aniline-diphenylamine phosphate reagent, and maintained at 85 °C for 10 min.

The hydrolysis of lactose in milk was measured by mixing 500 μL of purified lacZBa (8 U/mL) and commercial skim milk containing 5 g/100 mL of lactose in incubation at 45 °C for different times (0, 30, 60, 120 and 240 min). The reaction was stopped by incubating the reaction mixture in boiling water bath for 5 min. After deproteinization with trichloroacetic acid (10%) at 4 °C for 3 h, the supernatant was collected by centrifugation at 12,000 rpm for 15 min [32].

The lactose in commercial milk and its hydrolysis products (glucose and galactose) was analyzed by TLC, and quantified by HPLC. HPLC analysis was performed on Waters alliance 2695 separation module connected with 2414 refractive index detector using NH2P-504E column (5 μm, 4.6 × 250 mm, Shodex, Japan) eluted with 75% acetonitrile (1 mL/min). The concentration of glucose in the hydrolysis products was quantified by using an SBA-40D biological sensing analyzer, a kind of glucose biosensor (Biology Institute of the Shandong Academy of Sciences, Jinan, China) [33].

### 2.10. Statistical Analysis

All assays were performed at least three times, and the results were expressed as the mean ± standard deviation of these replicates. Statistical analysis was performed with Student’s *t*-test. *p* values of <0.01 were considered statistically significant.

### 2.11. Bioinformatic Analysis

Multiple sequence alignment was carried out by using Clustal Omega, and displayed by ESPript 3.0. The phylogenetic tree was constructed in the MEGA 7 package using the maximum likelihood (ML) method. ProtParam tool of ExPASy was used to predict the Mw (molecular weight) and theoretical pI (isoelectric point) of the lacZBa [1].

The SWISS-MODEL server (https://swissmodel.expasy.org/) (accessed in 2 April 2020) was used to develop a homology model for lacZBa. The crystal structure of β-galactosidase (PDB 3tts.1) from *Bacillus circulans* sp. alkalophilus was used as the template. The structure with the highest GMQE score (Global Model Quality Estimate) was chosen for further structure refinement. The model structure cleaning, and minimization was carried out by using Yasara software (Yasara Biosciences GmbH, Vienna, Austria). The 3D structure of lactose (ligand) was drawn in ChemDraw 18.0. After the ligand and the receptor preparation, docking runs was performed using Yasara software, and the docking parameters is shown in Table S1. The docking results were analyzed using Yasara software, LigPlot^+^ V2.2 (Cambridge, UK), and BIOVIA Discovery Studio Visualizer 2020 (San Diego, CA, USA) [34].

## 3. Results and Discussion

### 3.1. Cloning and Sequence Analysis of lacZBa

We isolated the novel bacterium *B. aryabhattai* Gel-09. Following genome data analyses, we found a hypothetical β-galactosidase protein, lacZBa. For a more in-depth study, we cloned and overexpressed the encoding gene *lacZBa* in *E. coli*. The length of the *lacZBa* open reading frame was 1950 bp, and the lacZBa protein consisted of 649 amino acids (AAs). The MW (molecular weight) and theoretical pI (isoelectric point) of lacZBa were 75.42 kDa and 5.38, respectively.

**Figure 1:**
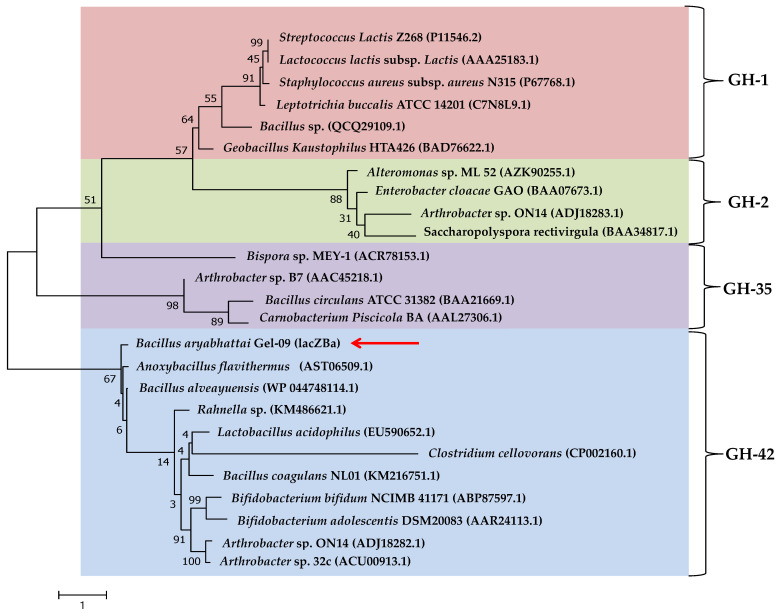
Evolutionary tree of β-galactosidases AA sequences based on the maximum likelihood (ML) method. Bootstrap values were expressed as a percentage of 500 replicates. The position of lacZBa in the phylogenetic tree was marked by the red arrow.

**Figure 2:**
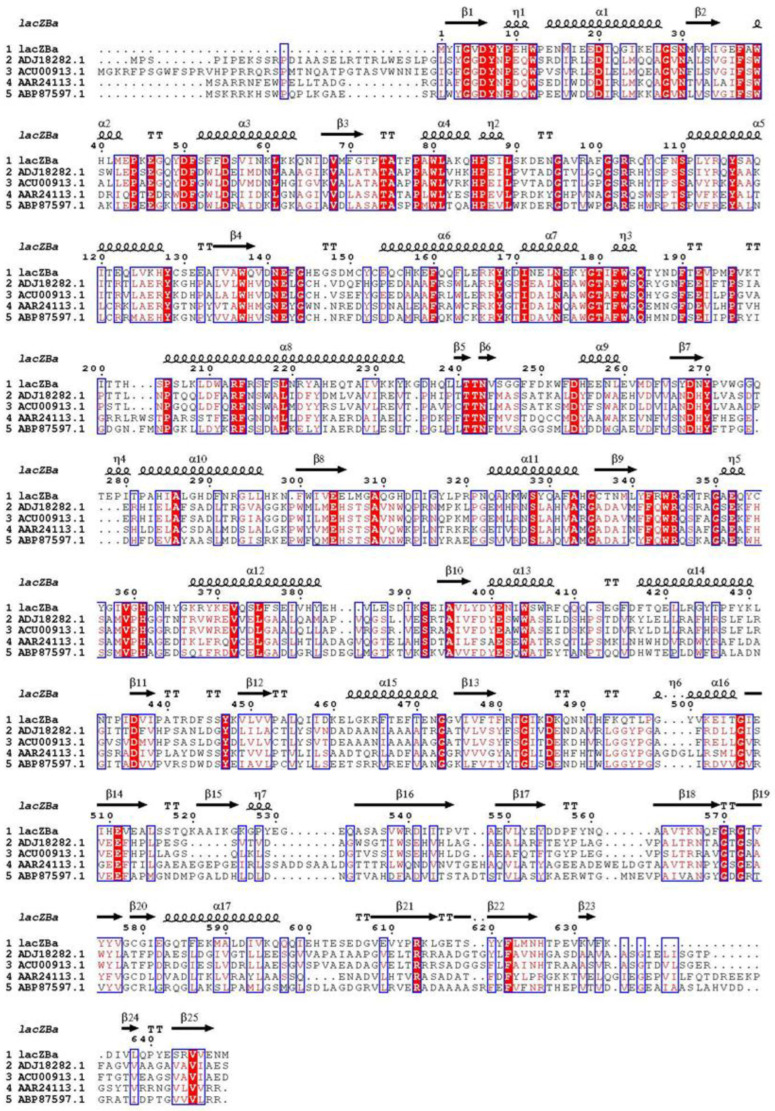
Comparison of the sequence of lacZBa with those of related β-galactosidases from GH-42. Highly conserved residues were labeled red. The accession numbers of β-galactosidase are as follows: *Bifidobacterium adolescentis* DSM20083(AAR24113.1), *Arthrobacter* sp. ON14(ADJ18282.1), *Arthrobacter* sp. 32c(ACU00913.1), *Bifidobacterium bifidum* NCIMB 41171(ABP87597.1).

BLAST analysis showed that lacZBa had the highest similarity (99.85%) with β-galactosidase (WP_182028680.1) of *Bacillus* sp. ME75. To verify the phylogenetic position of lacZBa among the characterized β-galactosidases, we used the maximum likelihood method to construct a phylogenetic tree of lacZBa and 24 β-galactosidases, which have been reported from different glycoside hydrolases (GH) families (Figure 1). The results showed that lacZBa was in clade 42, formed from GH-42 (Figure 1), whereas clade 1, clade 2, and clade 35 were formed by β-galactosidases from GH-1, GH-2, and GH-35, respectively. Moreover, lacZBa had different sequence identity with β-galactosidases from *Bacillus alveayuensis*, *Anoxybacillus flavithermus*, *Bacillus coagulans* NC01, and *Lactobacillus acidophilus*, exhibiting 72.4%, 68.2%, 26.7%, and 24.3% identities, respectively. lacZBa was considered a new member of the GH-42. Sequence and phylogenetic analyses indicated that each GH family appeared to be genealogically related to each other through a separate gene lineage, because the four families are distantly related to each other [35]. In addition, we used multiple sequence alignment to compare lacZBa with other related GH-42 β-galactosidase from different bacteria, and the conserved sequence and catalytic domain were further analyzed (Figure 2). The results showed that GH-42 β-galactosidase had conserved amino acid residues and a catalytic domain. β-galactosidases from the four families belonged to the GH superfamily A and had a typical (β/α)_8_-barrel structure. Members of the GH-42 cleave glycosidic bonds via retaining mechanisms, in which the product maintained the same conformation as the original substrate. Two glutamate or aspartic acid residues played an important role in catalysis; one acted as an acid-base catalyst, and the other acted as a nucleophilic reagent. The catalytic process consisted of a two-step double substitution mechanism [36].

**Figure 3:**
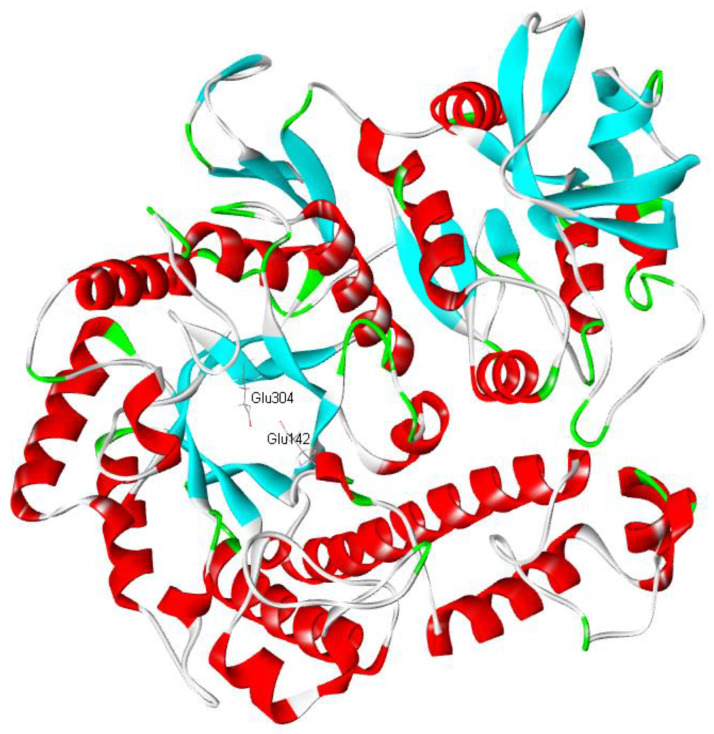
Three-dimensional schematic diagram of the folded structure of the (β/α)_8_-TIM barrel of lacZBa.

The crystal structure of β-galactosidase (PDB 3tts.1) from *Bacillus circulans* sp. *alkalophilus* was used as the template to simulate the lacZBa structure. Moreover, the ramachandran plots of the structure was also analyzed (Figure S1). The predicted 3D structure of lacZBa fit well with the typical (β/α)_8_ TIM barrel scaffold. Two conserved catalytic residues Glu142 and Glu304 were observed in the substrate binding pocket in the TIM barrel of lacZBa (Figure 3), which were similar to Glu141 and Glu312 of β-galactosidases from *Thermotoga naphthophila* RUK-10 [37] and *T. thermophilus* A4. β-galactosidase A4-β-Gal from *T. thermophilus* A4 is one of the thermostable members of GH-42, and it is the first GH-42 enzyme to have its crystal structure determined. A4-β-Gal possesses a (β/α)_8_ TIM barrel structure [17].

### 3.2. Heterologous Expression and Purification of lacZBa

**Figure 4:**
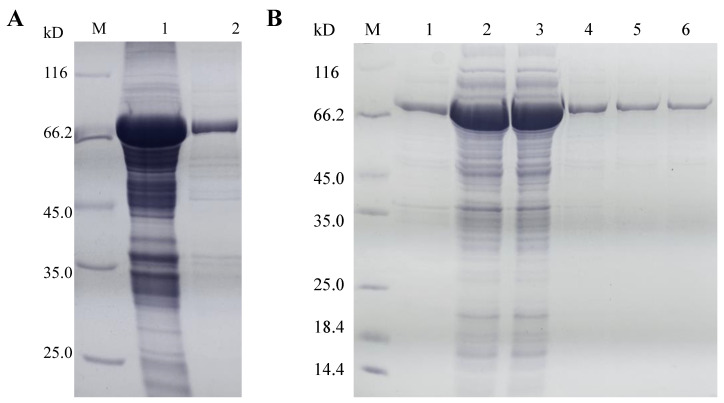
SDS-PAGE analysis of lacZBa. (**A**) Lanes: M, protein standard; 1, Precipitate fraction of lacZBa; 2, Supernatant fraction of lacZBa; (**B**) Lanes: M, protein standard; 1, Supernatant fraction of lacZBa; 2, Supernatant fraction of lacZBa after salting with 40% saturated ammonium sulfate solution; 3, Supernatant fraction of lacZBa after dialysis; 4,5,6, Supernatant fraction of lacZBa after anion-exchange chromatography with different gradients.

To investigate its biochemical properties, we expressed lacZBa in *E. coli* BL21(DE3). Cells were cultured in LB medium for 6–8 h, and seeded into TB medium at 5% (*v/v*) inoculum. The cells were induced by adding 0.2 mmol/L IPTG after 3 h of incubation in TB medium, and continued to be cultured for 36 h. After fermentation, the optical density (OD_600_) reached 22.14. Additionally, there was significant lacZBa activity in the recombinant cell lysates. Because lacZBa is an intracellular enzyme, the recombinant cells were disrupted by ultrasonic treatment, and the soluble protein fractions of the lysed cells were collected as crude enzyme. The crude enzyme was purified by ammonium sulfate precipitation and dialysis following DEAE-μSphere anion-exchange chromatography (Table 1). SDS-PAGE analysis showed that the subunit molecular mass of the purified protein was ~75 kDa (Figure 4). By comparison, most β-galactosidases belonging to the same GH family had a similar number of AA residues. Most members of GH-42 contain more than 600 AA residues in each monomer. For example, *B. bifidum* NCIMB 41171 [38] β-galactosidase contains 689 AA residues, *B. coagulans* β-galactosidase contains 665 AA residues, and *B. aryabhattai* Gel-09 β-galactosidase (lacZBa) contains 649 AA residues. Members of GH-2 contain more AA residues than those of GH-42, including β-galactosidase from *Saccharopolyspora rectivirgula* [39] (1251 AA residues), β-galactosidase from *Alteromonas* sp. ML52 [40] (1059 AA residues), and β-galactosidase from *Enterobacter cloacae* GAO [41] (1028 AA residues). β-galactosidases from GH-35 and GH-1 contain a smaller number of AA residues. β-galactosidase from *Bacillus circulans* (GH-35) contains 586 AA residues, while β-galactosidase from *Leptotrichia buccalis* ATCC 14201 (GH-1) [42] only has 467 AA residues, and its subunit molecular mass is ~53 kDa.

### 3.3. Effects of pH and Temperature on lacZBa

**Figure 5:**
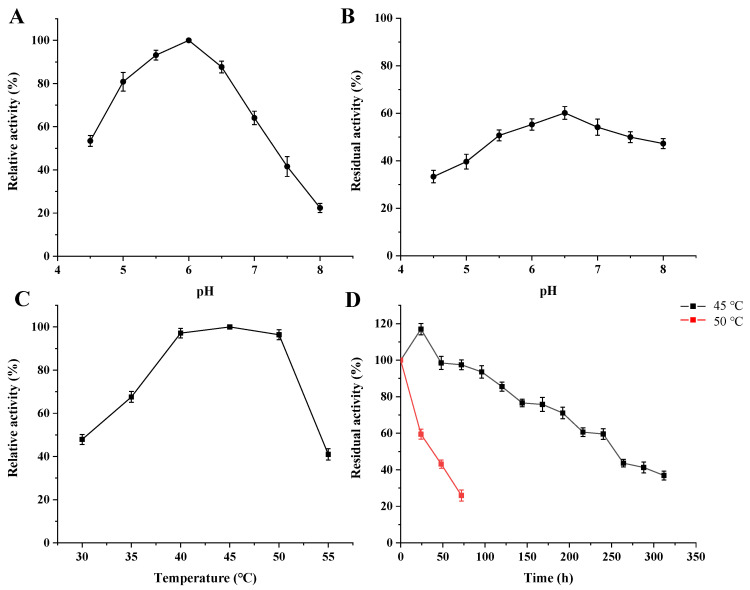
Effect of pH and temperature on lacZBa activity using oNPG as substrates. (**A**) Effect of pH from 4.5 to 8.0 on the lacZBa activity. (**B**) The pH stability of lacZBa incubated at pH 4.5–8.0 for 24 h. (**C**) Effect of temperature from 30 to 55 °C on the lacZBa activity. (**D**) The thermostability of lacZBa incubated at 45 and 50 °C for several hours.

Figure 5A shows that lacZBa had its highest activity at pH 6.0, with >60% of its maximum activity at pH 5.0–7.0. However, its activity decreased sharply at pH > 7.0. In addition, lacZBa was stable at pH 5.5–7.0 and retained more than 50% activity following incubation at pH 5.5–7.0 for 24 h (Figure 5B). Therefore, lacZBa is suitable for applications in neutral or weakly acidic conditions, which are suitable for lactose hydrolysis in milk (pH 6.0–7.0) [43].

These results reveal that the optimal temperature of lacZBa is 45 °C, with 97.1% and 96.3% relative activities at 40 °C and 50 °C, respectively (Figure 5C). However, the enzyme exhibits 47% of its maximum activity at 55 °C. β-galactosidases have an optimum temperature range of 0–50 °C in *Arthrobacter* sp. 32c [44] and 4–35 °C in *Rahnella* sp. R3 [45]. Psychrophilic Antarctic *Planococcus* isolate [46] contains cold-adapted enzymes. β-galactosidases derived from *B. coagulans* NL01 (50–65 °C) [43] and *Thermotoga naphthophila* RUK-10 (70–90 °C) [25] exhibit high enzymatic activity at high temperatures. In our study, lacZBa exhibited the highest activity at a relatively mild temperature. Therefore, lacZBa is a typical mesophilic enzyme.

### 3.4. Thermostability of lacZBa

Based on its sequence homology, lacZBa belongs to GH-42. Several GH-42 β-galactosidases are derived from extremophiles such as thermophilic, cryophilic, and halophilic microbes, and most GH-42 β-galactosidases have good thermostability. When evaluating the thermostability of lacZBa, we found that lacZBa could be activated following incubation at 45 °C for several minutes. Moreover, the activity of the purified enzyme was ~117% following storage at 45 °C for 24 h and 97% following storage at 45 °C after 100 h. Moreover, the half-life of lacZBa was 264 h at 45 °C and 36 h at 50 °C (Figure 5D). Therefore, lacZBa is not only thermally activated at the optimum temperature, but has good thermostability at 45–50 °C. Additionally, lacZBa has better thermostability than other GH-42 β-galactosidases, which have similar temperature range as lacZBa (Table 2). In fact, it has been reported that β-galactosidase from *Rahnella* sp. R3 loses ~70% of its activity following incubation at 45 °C for 15 min [47] and is fully inactivated after 30 min at 45 °C. Furthermore, β-galactosidase from the psychrotrophic and halotolerant *Planococcus* sp. L4 [48] is fully inactivated after 10 min at 45 °C.

### 3.5. Effect of Metal Ions and EDTA on lacZBa Activity

Different concentrations of EDTA significantly inhibited lacZBa activity, indicating that the catalytic action of lacZBa required the presence of divalent cations (Table 3). Furthermore, the addition of 1 mM Ca^2+^, Mg^2+^, and Fe^2+^ moderately improved enzymatic activity (109–127%). Mn^2+^, Zn^2+^, Co^2+^, and higher concentrations (5 mM) of Ca^2+^, Mg^2+^, Fe^2+^ significantly enhanced enzyme activity (150%). In contrast, enzymatic activity was inhibited by Cu^2+^. Therefore, the most common divalent cations are unable to inhibit the activity of lacZBa, and lacZBa is suitable in dairy products where the mineral concentration is relatively high and the pH of milk ranges between 6 and 7. Additionally, we have completed with the effects of anionic groups including 1 mM and 5 mM Cl^−^, 2 mM and 10 mM Cl^−^, 1 mM and 5 mM SO_4_ ^2−^ on lacZBa activity. The results showed that anionic groups did not significantly promote or inhibit on lacZBa activity.

Previous studies have shown that Mg^2+^ was beneficial for the activity of most β-galactosidases [43], regardless of the GH family. β-Galactosidases from *Alteromonas* sp. ML117 [50], *Paracoccus* sp. 32d [51], and *Planococcus* sp. L4 [49] are activated by Mg^2+^. Conversely, Cu^2+^ inhibits the activity of most β-galactosidases including β-galactosidases from *Arthrobacter* sp. ON14 [18], *Arthrobacter* sp. 20B [52], and *B. aryabhattai* GEL-09 (this study). Unlike lacZBa, some β-galactosidases like YesZ from *Bacillus subtilis* [10] and Am0874 from *Akkermansia muciniphila* [21] do not require to be activated by extra metal cations. Instead, the activities of YesZ and Am0874 show no alteration in the presence of EDTA. It is noteworthy that lacZBa can be activated by Ca^2+^, which has is greatly beneficial for its use in the dairy industry. However, Ca^2+^ had no influence on β-galactosidase (GalA) from *Arthrobacter* sp. ON14 [18].

### 3.6. Kinetic Parameters of lacZBa

Kinetic analysis showed that the Michaelis constant (*K*_m_) was 14.38 mM for oNPG and 85.09 mM for lactose (Table 4). At 45 °C, the *K*_m_ values were six times lower for oNPG than for lactose. The maximum rate (*V*_max_) was 1.75 and 14.66 U/mg when lactose and oNPG were used as substrate, respectively. Similar to lacZBa, β-galactosidase (Bca-β-gal) from *Bacillus circulans* sp. *alkalophilus* has higher affinity for oNPG [16]. However, R-β-gal, which belongs to GH-42 and is derived from *Rahnella* sp. R3, has a higher substrate affinity towards lactose than oNPG. Its *K*_m_ is three to 10 times higher for oNPG than for lactose [47].

### 3.7. Docking Analysis of lacZBa

**Figure 6:**
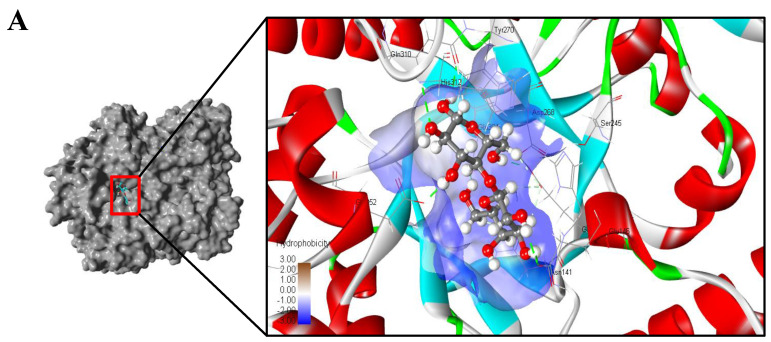

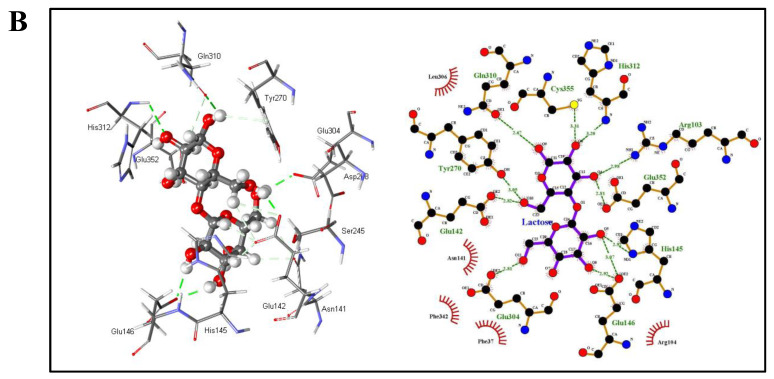
Molecular docking of lacZBa with lactose. (**A**) Overall structure and substrate binding pocket analysis of lacZBa. (**B**) Schematic diagram of lacZBa/lactose interactions.

Lactose was docked into the substrate binding pocket of lacZBa to generate the binding mode. The docking results revealed that lactose binds to the active site pocket of lacZBa (Figure 6A). To reveal the substrate recognition mechanisms, substrate–enzyme interaction analyses was performed. We assayed the hydrogen bonding networks and hydrophobic interactions at the enzyme active pocket (Figure 6B). Ten AA residues (Arg103, Glu142, His145, Glu146, Tyr270, Glu304, Gln310, His312, Glu352, and Cys355) formed 11 hydrogen bonds with lactose. There were hydrophobic interactions between lactose and residues Phe37, Arg104, Asn141, Leu306, and Phe342. These results showed that lacZBa has a strong lactose binding ability, which is beneficial to substrate hydrolysis. There were one hydrophobic interaction and seven H-bonds for glucose residues and three hydrophobic interactions and four H-bonds for galactose residues (Figure 6B). The binding energy between lacZBa and lactose was −105.3052 kcal/mol, which was lower than that of the binding energy (−93.5294 kcal/mol) between *B. circulans* (PDB: 3tts.1) and lactose.

In addition, molecular docking was used to analyze the interaction between lactose and catalytic residues (Glu142 and Glu304). The carboxyl groups of Glu142 and Glu304 located on the hydrophilic face of the glucose residue and hydrophobic face of the galactose residue, respectively. Based on multiple sequence alignment (Figure 2) and molecular docking analysis (Figure 6B), Glu142 probably acted as the nucleophile (bases), and Glu304 likely acted as the proton donor (acids).

### 3.8. Hydrolysis of Lactose in Lactose Solution and Commercial Milk

Lactose is a unique carbohydrate present in milk. Human milk contains ~7.4% lactose, which is the primary source of energy to newborns. The lactose content of commercially available cow milk is ~4.8%. In East Asia, more than 90% of people are unable to digest lactose, and may develop symptoms of lactose intolerance. β-Galactosidases, which have been widely used to hydrolyze lactose in dairy products, are beneficial for lactose-intolerant people.

**Figure 7:**
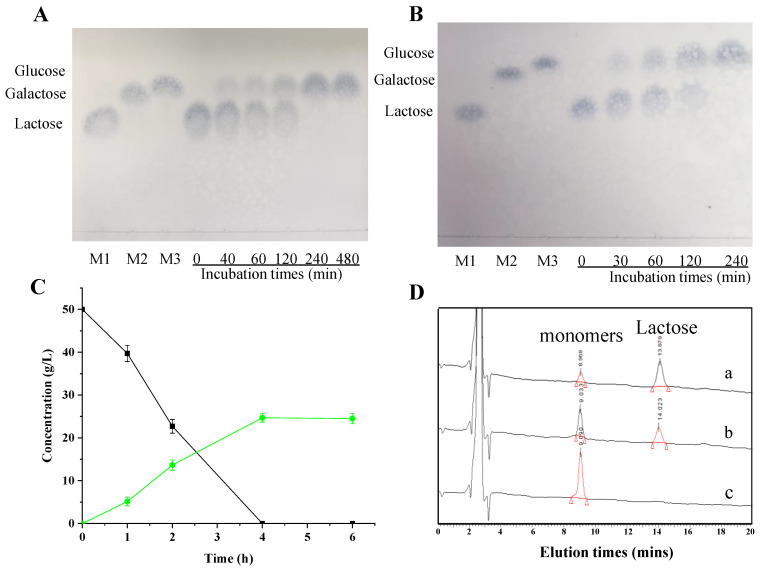
Analysis of lactose hydrolysis ability of lacZBa in lactose solution and commercial milk. (**A**) TLC analysis of lactose solution hydrolyzing by lacZBa. Standard samples: (M1) lactose, (M2) galactose, (M3) glucose. (**B**) TLC analysis of commercial milk hydrolyzing by lacZBa. (**C**) HPLC-RID profiles of commercial milk hydrolyzing by lacZBa for 0, 1, 2, 4, 6 h. (■) Concentration of lactose, (●) concentration of glucose. (**D**) HPLC-RID profiles of commercial milk hydrolyzing by lacZBa for 1 h (a), 2 h (b), 4 h (c).

To determine the lactose hydrolysis ability of lacZBa, 8 U/mL lacZBa was used to hydrolyze a 50 g/L lactose solution. The hydrolytic products of lacZBa were analyzed by TLC. The results showed that lactose could be completely hydrolyzed by lacZBa within 4 h (Figure 7A). To further investigate the application performance of lacZBa, we used commercial milk. We mixed 8 U/mL lacZBa with commercial skim milk, and samples were taken at different time periods. After deproteinization of commercial milk samples with 10% trichloroacetic acid at 4 °C for 3 h, we measured the hydrolytic products by TLC, glucose biosensor and HPLC. As shown in Figure 7B, lactose in commercial milk was completely hydrolyzed within 4 h, indicating that lacZBa was sufficient. Lactose in commercial milk was progressively hydrolyzed as the reaction progressed, and 54.58% lactose was hydrolyzed in the first 2 h (Figure 7C). Lactose was completely hydrolyzed by lacZBa when the reaction time was extended to 4 h (Figure 7C,D). However, no galactooligosaccharides (GOS) was detected during the hydrolysis process.

Even though several β-galactosidases have been heterologously expressed and identified, only a few are available for commercial applications [53]. Currently, the food industry utilizes β-galactosidase from *Saccharomyces cerevisiae* and *Aspergillus oryzae* [54], both of which have relatively good properties, including an optimum pH for breaking down lactose in milk (6.0–7.0). lacZBa has the best activity at pH 6.0 and is stable at pH values between 6.0 and 7.0, which is compatible with the application of lacZBa in dairy products. At the same time, thermostability is another important limitation in enzyme applications. In lactose hydrolysis, the maximal degradation rate was 100% within 4 h using lacZBa. In this study, lacZBa can maintain stability at 45 °C for more than 200 h, which makes lacZBa suitable for industrial applications. Although thermostable β-galactosidases have significant advantages in the production of low-lactose dairy products [55], experimental data supporting lactose hydrolysis by GH-42 β-galactosidases are absent or weak [56]. Additionally, not all the β-galactosidases are able to hydrolyze lactose [57]. Therefore, the properties of lacZBa may provide support for future scientific research on GH-42 β-galactosidases.

## 4. Conclusions

In this study, we expressed and characterized a novel β-galactosidase lacZBa from *B. aryabhattai* Gel-09. Based on protein sequence alignment and phylogenetic analyses, lacZBa was determined to be a member of GH-42. The activity of the enzyme could be thermally activated and exhibited excellent thermal stability at 45–50 °C. Furthermore, molecular docking and hydrolysis experiments showed that lacZBa could efficiently recognize and catalyze lactose. Notably, lactose in milk was completely hydrolyzed within 4 h following addition of 8 U/mL of lacZBa at 45 °C, suggesting that it is a promising candidate for further use and research in the dairy industry. However, lacZBa has limited activity toward lactose, and the affinity of lacZBa for lactose as a substrate is not as good as that of oNPG. In future study, we are going to design a series of mutants of lacZBa, which can be better applied to the production of lactose-free milk by screening mutants of lacZBa with high hydrolysis performance. Moreover, we will try to heterologously express the protein in a generally recognized as safe host and optimize its enzyme activity for a better application in food industry.

## Figures and Tables

**Table 1:** Purification scheme of lacZBa.

Purification Steps	Total Protein (mg)	Total Activity (U)	Specific Activity (U/mg^−1^)	Purification (-Fold)	Yield (%)
Crude Enzyme	190.2	1988.1	10.45	1	100
Ammonium Sulfate Fraction	54.6	653.6	11.97	1.14	32.86
Anion-exchange Chromatography	12.0	291.5	15.98	1.52	14.65

**Table 2:** Comparison of the properties of GH-42 β-galactosidase from different microbial sources.

Microorganism	Molecular Mass (kDa)	Thermal Stability		Optimum Temperature (°C)	Optimum pH	References
		Time (min/h)	Residual Activity (%)			
*B. aryabhattai*	NR (75) ^a^	36 h (50 °C)	50	45	6.0–7.0	This study
		264 h (45 °C)	50			
*G. stearothermophilus*	NR (70) ^a^	10 min (70 °C)	0	55	6.5	[17]
*B. bifidum* NCIMB41171	Bbg I: 875 (160) ^a^ Bbg II: 178 (80) ^a^ Bbg III: 351 (190) ^a^ Bbg IV: 249 (131) ^a^	3 h (40 °C)	80	50	5.4–5.8 (BbgII) 6.4–6.8	[38]
		20 min (55 °C)	20			
*B. coagulans* NL01	NR (76.04) ^a^	3.5 h (60 °C)	50	55–60	5.5–6.5	[43]
*Arthrobacter* sp. 32c	195.5 (75.9) ^a^	NR	NR	50	6.5–8.5	[44]
*Rahnella* sp. R3	225 (77.1) ^a^	15 min (45 °C)	30	35	6.5	[45]
*Planococcus* sp. L4	NR (78) ^a^	10 min (45 °C)	0	20	6.8	[49]
*Thermotoga naphthophila* RUK-10	130–140 (70) ^a^	10.5 h (75 °C)	50	90	6.8	[37]
*Arthrobacter* sp. ON14	116	NR	NR	37	8.0	[18]

NR, not reported; ^a^ Molecular mass of the monomer.

**Table 3:** Effect of metallic cations and EDTA on lacZBa activity.

Metallic Cations and EDTA	Relative Activity (%)	
	1 mM	5 mM
Control	100 ± 0.4	100 ± 0.3
Mn^2+^	144 ± 2.8	181 ± 1.5
Zn^2+^	151 ± 1.4	157 ± 2.6
Cu^2+^	0	0
Co^2+^	169 ± 1.3	183 ± 0.7
Fe^2+^	125 ± 2.1	150 ± 2.8
Mg^2+^	122 ± 3.3	164 ± 1.4
Ca^2+^	107 ± 2.8	157 ± 2.8
EDTA	49 ± 1.3	51 ± 1.8

**Table 4:** Kinetic parameters for the purified lacZBa.

Substrate	*K*_m_ (mM)	*V*_max_ (U/mg)	*K*_cat_ (s^−1^)	*k*_cat_/*K*_m_ (s^−1^ mM^−1^)
oNPG	14.38 ± 0.65	14.66 ± 0.59	53.73 ± 2.79	3.74 ± 0.16
Lactose	85.09 ± 3.40	1.75 ± 0.09	6.56 ± 0.27	0.07 ± 0.003

## Data Availability

Data are contained within the article.

## Supplementary Materials

The following supporting information can be downloaded at: https://www.mdpi.com/article/10.3390/foods11030372/s1, Table S1: Docking parameters for receptor (lacZBa) and lactose (ligand); Figure S1: Ramanchandran plots of lacZBa structure model.
