# Supplementary material for: A Novel Thermal-Activated β-Galactosidase from Bacillus aryabhattai GEL-09 for Lactose Hydrolysis in Milk
Source: Foods. 2022 Jan 27;11(3):372. doi: 10.3390/foods11030372 (PMC8834341; doi:10.3390/foods11030372)
Supplement: Supplementary file 1 [file foods-11-00372-s001.zip › foods-1490420-supplementary.pdf]

Table S1. Docking parameters for receptor (lacZBa) and lactose (ligand).

Parameteres	Value
X coordinate of the center 190.2	15.95041
Y coordinate of the center	44.9083
Z coordinate of the center	-4.28244
Size in the radius	8.578 Å
Maximum number of binding modes to generate	100

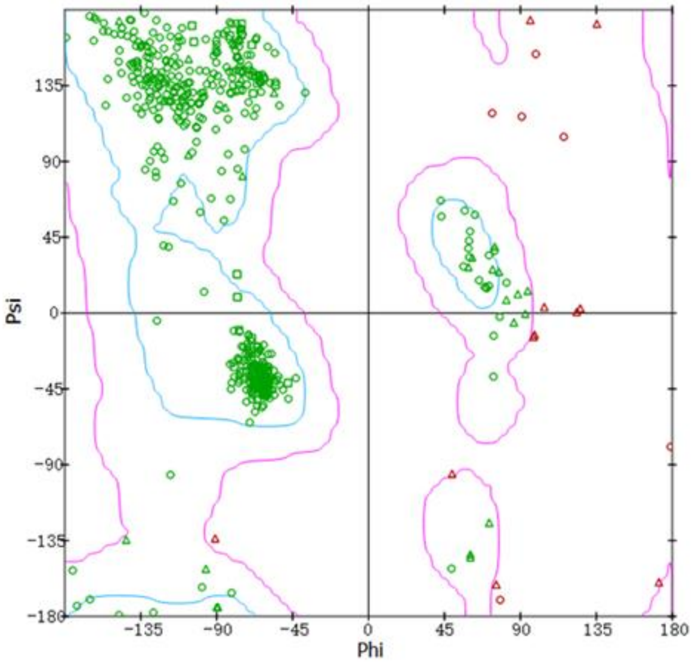

Figure S1. Ramanchandran plots of lacZBa structure model.
